# Respiratory Versus Gastrointestinal Malignancies: Systemic Inflammation, Cardiovascular Burden, and All-Cause Mortality

**DOI:** 10.3390/jcm15145752

**Published:** 2026-07-22

**Authors:** Bozhidar Krastev, Natalia Spasova, Petranka Troyanova, Elena Kinova, Assen Goudev

**Affiliations:** 1 Department of Emergency Medicine, Medical University of Sofia, 1527 Sofia, Bulgaria; spasova.natalia@gmail.com (N.S.); kinova.e@abv.bg (E.K.); agoudev@abv.bg (A.G.); 2 Clinic of Cardiology, University Hospital “Tsaritsa Yoanna-ISUL”, 1527 Sofia, Bulgaria; 3 Department of Medical Oncology, Medical University of Sofia, University Hospital “Tsaritsa Yoanna-ISUL”, 1527 Sofia, Bulgaria; p_troyanova@abv.bg

**Keywords:** systemic immune-inflammation index, SII, cancer, mortality, tumor stage, metastasis, cardiovascular burden

## Abstract

**Background/Objectives:** Systemic inflammation is common in patients with cancer and may reflect tumor activity, disease extent, and the patient’s overall clinical condition. The systemic immune-inflammation index (SII), calculated from neutrophil, platelet, and lymphocyte counts, is a simple marker, but its relationship with tumor type, cardiovascular burden, and mortality is not fully clarified. To compare patients with respiratory system malignancies (RSM) and gastrointestinal tract malignancies (GITM) in terms of cardiovascular comorbidity, inflammatory profile, tumor-related characteristics, and recorded all-cause mortality, and to assess the association between SII and mortality. **Methods:** We analyzed 879 patients with solid malignancies, including 350 with RSM and 529 with GITM. SII was calculated as platelet count × neutrophil count/lymphocyte count. Cardiometabolic and cardiovascular burden was assessed according to the number of recorded cardiometabolic risk factors and cardiovascular diseases. The main outcome was recorded all-cause mortality. **Results:** Patients with RSM were younger and more often male, and more frequently had stage IV disease, whereas patients with GITM had a more pronounced cardiometabolic risk profile. Mortality was higher in RSM than in GITM (42.3% vs. 29.9%), and SII was also higher in RSM (median 1117.8 vs. 716.8). In the overall cohort, higher SII was associated with mortality after adjustment for age, sex, cancer type, stage, and metastatic disease (OR 1.11 per 1000-unit increase, 95% CI 1.02–1.21; *p* = 0.013). Mortality increased across SII tertiles from 22.5% to 34.5% and 47.4%. Adding SII to the clinical model led to only a small increase in AUC, from 0.719 to 0.729. **Conclusions:** SII was higher in patients with RSM and was associated with recorded all-cause mortality. However, its added prognostic value was modest. SII should therefore be interpreted as a supportive inflammatory marker, together with tumor stage, metastatic disease, and the broader clinical condition of the patient.

## 1. Introduction

Cancer and cardiovascular disease often coexist in the same patient. In many cases, this coexistence is not solely a result of older age or cancer treatment. A considerable proportion of patients with malignancy already have cardiometabolic risk factors, including smoking, arterial hypertension, diabetes mellitus, obesity, dyslipidemia, alcohol exposure, and chronic low-grade inflammation. These factors may contribute to both cancer development and cardiovascular morbidity and may also influence the interpretation of inflammatory biomarkers [1,2].

This becomes particularly relevant when respiratory and gastrointestinal malignancies are compared. Both tumor groups account for a large proportion of the global cancer burden but differ in several clinical and etiological aspects [3]. Respiratory cancers are strongly linked to smoking, chronic pulmonary inflammation, air pollution and occupational or environmental exposures. Gastrointestinal cancers are more heterogeneous and are often associated with metabolic disorders, obesity, alcohol use, dietary factors, chronic gastrointestinal inflammation, infection-related mechanisms and other local or systemic pathways. As a result, the two tumor groups may share some risk factors, yet the cardiovascular and inflammatory background of the patients often differs.

In daily oncology practice, systemic inflammation is rarely explained by the tumor alone. It may reflect advanced disease, metastatic spread, infection, nutritional deterioration, impaired immune response, or pre-existing cardiovascular and metabolic disease. For this reason, inflammatory indices derived from routine blood tests should be interpreted together with the clinical profile of the patient, rather than as isolated cancer-specific markers.

The systemic immune-inflammation index (SII) is calculated as platelet count × neutrophil count/lymphocyte count. It is simple, inexpensive and based on routinely available complete blood count parameters. From a biological point of view, SII combines several relevant signals: neutrophilia as a marker of systemic inflammation, thrombocytosis as a marker of platelet activation and thrombo-inflammatory activity, and lymphopenia as a possible marker of impaired immune reserve [4]. Previous studies have shown that higher SII is associated with worse outcomes in different solid tumors, including lung, colorectal and gastric cancer [5,6,7,8]. Its prognostic value, however, appears to depend on tumor type, disease stage, metastatic status and the overall cardiovascular and metabolic burden of the patient [9,10].

Based on this background, the comparison between respiratory and gastrointestinal malignancies may provide useful information about the relation between tumor type, cardiovascular comorbidity, systemic inflammation, and outcome. The aim of the present study was to compare these two tumor groups with respect to cardiovascular comorbidity, systemic immune-inflammatory profile, tumor-related characteristics, and recorded all-cause mortality, and to evaluate the role of SII as a readily available marker of systemic inflammation in a real-world oncology cohort.

## 2. Materials and Methods

### 2.1. Study Design and Population

This retrospective observational study included consecutive hospitalized adult patients with respiratory or gastrointestinal solid malignancies. Patients were hospitalized between September 2023 and December 2025 at the Oncology Clinic of University Hospital “Tsaritsa Yoanna—ISUL”, Sofia, Bulgaria. For the present analysis, patients were classified into two tumor-type cohorts, defined as respiratory system malignancies (RSM) and gastrointestinal tract malignancies (GITM), respectively.

During the study period, 2020 consecutive hospitalized adult patients aged ≥18 years with solid malignancies were screened. Because many patients were hospitalized repeatedly during ongoing oncological treatment, each patient was included only once, using clinical and laboratory data from the first hospitalization before administration of the first cycle of systemic anticancer therapy. For the present analysis, patients with respiratory system malignancies or gastrointestinal tract malignancies were selected, as these represented the most frequent tumor groups treated at our institution. The remaining screened patients had solid malignancies at other anatomical sites, including prostate, breast, and other tumor locations, and were not included in the present analysis. The final analytical cohort included 879 unique patients, comprising 350 patients with RSM and 529 patients with GITM.

Eligibility for the present analysis required ascertainable recorded mortality status and available baseline neutrophil, lymphocyte, and platelet counts necessary for calculation of SII. All 879 patients included in the analytical cohort met these requirements. No otherwise eligible patient was excluded because of missing mortality status or missing baseline hematological parameters.

The RSM group included malignancies of the larynx, bronchi, and lung. For this reason, the term “respiratory system malignancies” was used rather than “lung cancer.” The GITM group included gastric, colorectal, hepatocellular, and pancreatic carcinomas, as well as other gastrointestinal malignancies. The study was designed to compare these broad organ-system groups rather than individual tumor sites or histological subtypes.

Demographic data, cancer-related characteristics, cardiovascular comorbidities, cardiometabolic risk factors, laboratory results, and outcome status were extracted from hospital electronic medical records. Cancer stage and metastatic disease status were recorded from the available clinical documentation.

### 2.2. Clinical Variables

Baseline clinical and cardiovascular variables included age, sex, metastatic disease, cancer stage, coronary artery disease, previous myocardial infarction, previous stroke, diabetes mellitus, hypertension, obesity, atrial fibrillation, dyslipidemia, vascular disease, valvular disease, heart failure, smoking, alcohol use, pulmonary embolism, asymptomatic pulmonary embolism and deep vein thrombosis.

Cancer stage was categorized as stage I, II, III, or IV. Metastatic disease was analyzed as a separate binary variable. Cancer stage and metastatic disease status were extracted as documented in the medical records. Stage IV disease and metastatic disease were analyzed as separate variables, because the latter reflected explicitly documented distant metastatic involvement. Cardiovascular comorbidities and cardiometabolic risk factors were recorded as present or absent according to the available medical records. Obesity was recorded as a documented clinical diagnosis in the medical records and was analyzed as a binary variable (present/absent).

### 2.3. Laboratory Parameters and Inflammatory Index

Baseline laboratory parameters were obtained from hospital records during the first hospitalization before the initiation of treatment. The hematological parameters of interest were neutrophil count, lymphocyte count and platelet count. The systemic immune-inflammation index was calculated as platelet count × neutrophil count/lymphocyte count and was evaluated both as a continuous variable (per 1000-unit increase) and as a categorical variable using tertiles in sensitivity analyses.

### 2.4. Outcome Definition

The primary outcome was recorded all-cause mortality at the time of data extraction. Mortality status was ascertained through the National Health Insurance Fund database and hospital electronic medical records.

Because exact dates of death and complete time-to-event information were not available for all patients, mortality was analyzed as a binary outcome using logistic regression. Therefore, the results should be interpreted as associations with recorded all-cause mortality, not as time-to-event survival estimates.

### 2.5. Statistical Analysis

Continuous variables are summarized as median and interquartile range, and categorical variables as counts and percentages. Continuous variables were compared between RSM and GITM using the Mann–Whitney U test, and categorical variables using the chi-square test or Fisher’s exact test, as appropriate. No imputation of missing data was performed. For the few isolated missing values in other clinical variables, analyses were based on the available data, and the corresponding denominators are reported where applicable. The combined cardiometabolic and cardiovascular burden was defined as the total number of recorded diabetes mellitus, hypertension, obesity, dyslipidemia, smoking, alcohol use, coronary artery disease, previous myocardial infarction, previous stroke, atrial fibrillation, vascular disease, valvular disease, and heart failure cases. Differences in SII across cardiometabolic and cardiovascular burden categories were assessed using the Kruskal–Wallis test, and mortality across categories was compared using the chi-square test. Multivariable binary logistic regression models were used to evaluate factors associated with recorded all-cause mortality. Model 1 included age, sex, cancer type, cancer stage and metastatic disease; Model 2 included the same covariates with the addition of SII, entered as a continuous variable per 1000-unit increase, and results are presented as odds ratios with 95% confidence intervals. Model discrimination was assessed using the area under the receiver operating characteristic curve and calibration with the Hosmer–Lemeshow goodness-of-fit test. The incremental value of SII was examined by comparing nested models with and without SII using the likelihood-ratio test. As a sensitivity analysis, SII was also analyzed as a categorical variable using tertiles, with the lowest tertile as the reference category; patients were ranked according to baseline SII values and divided into three equal-sized tertiles, and this model was adjusted for age, sex, cancer type, cancer stage and metastatic disease. In an additional analysis, NLR was calculated as the absolute neutrophil count divided by the absolute lymphocyte count and evaluated as a continuous variable using the same multivariable model applied for SII. Its incremental value was assessed by likelihood-ratio testing, and the AUCs of the NLR and SII models were compared using bootstrap resampling. An interaction analysis was performed by including an interaction term between SII and cancer type in the multivariable model. All statistical tests were two-sided, and *p* < 0.05 was considered statistically significant.

Statistical analyses were performed using SPSS software, version 23.0.

### 2.6. Ethics

The study was conducted in accordance with the Declaration of Helsinki and was approved by the Institutional Ethics Committee of University Hospital “Tsaritsa Yoanna—ISUL”, Sofia, Bulgaria (Protocol No. 2/0801RS, dated 8 January 2026). Due to the retrospective design of the study and the use of anonymized clinical data, the requirement for written informed consent was waived.

## 3. Results

### 3.1. Study Population and Baseline Characteristics

Baseline characteristics according to cancer type are presented in Table 1. The final cohort included 879 patients, of whom 350 had RSM and 529 had GITM. The RSM cohort comprised laryngeal, bronchial, and pulmonary malignancies, whereas the GITM cohort included gastric, colorectal, hepatocellular, pancreatic, and other gastrointestinal malignancies. Patients with GITM were older than those with RSM, with a median age of 68.0 versus 62.5 years. In contrast, male sex was more common in the RSM group (80.6% vs. 57.3%). The two groups also differed in disease stage distribution. Stage IV disease was substantially more frequent among RSM patients (60.0% vs. 32.3%), whereas stages II and III were more common in GITM. Metastatic disease was numerically more frequent in GITM patients (27.0% vs. 21.1%), although this difference did not reach statistical significance. This finding should be interpreted cautiously, because metastatic disease was recorded only when explicitly documented and may therefore partly reflect differences in documentation within the retrospective records. Mortality was higher in the RSM group compared with the GITM group (42.3% vs. 29.9%).

Clinical characteristics according to cancer type are shown in Table 2. Overall, GITM patients had a more pronounced cardiometabolic risk profile, with higher rates of diabetes mellitus (19.5% vs. 9.4%), hypertension (60.5% vs. 49.1%), and obesity (21.7% vs. 14.3%). In contrast, stroke and smoking were more frequent in the RSM group, observed in 7.4% versus 2.8% and 49.4% versus 24.2% of patients, respectively. Deep vein thrombosis was more common among GITM patients (6.0% vs. 2.9%), while pulmonary embolism rates were similar between the two groups. No significant differences were observed for coronary artery disease, myocardial infarction, atrial fibrillation, dyslipidemia, vascular disease, valvular disease, heart failure, or alcohol use.

Laboratory parameters and inflammatory indices are summarized in Table 3. Patients with RSM showed a more pronounced inflammatory profile compared with patients with GITM. Neutrophil counts were higher in RSM patients, with median values of 5.9 versus 4.6, while lymphocyte counts were lower (1.5 vs. 1.7). Platelet counts were also higher in the RSM group (278.5 vs. 256.0). Consistent with these findings, the systemic immune-inflammation index was markedly higher in RSM compared with GITM, with median SII values of 1117.8 versus 716.8. All laboratory differences were statistically significant.

SII was similar across the cardiometabolic and cardiovascular burden categories, while mortality increased in patients with a higher overall burden (Supplementary Table S3).

### 3.2. Multivariable Logistic Regression Models for All-Cause Mortality

Multivariable logistic regression models for recorded all-cause mortality are presented in Table 4. In Model 1, which included age, sex, cancer type, stage and metastatic disease, male sex, GITM, stage IV disease and metastatic disease were associated with mortality. Male patients had higher odds of death than female patients, while GITM was associated with lower odds of mortality compared with RSM. Stage IV disease and metastatic disease were also independently associated with higher mortality.

**Figure 1:**
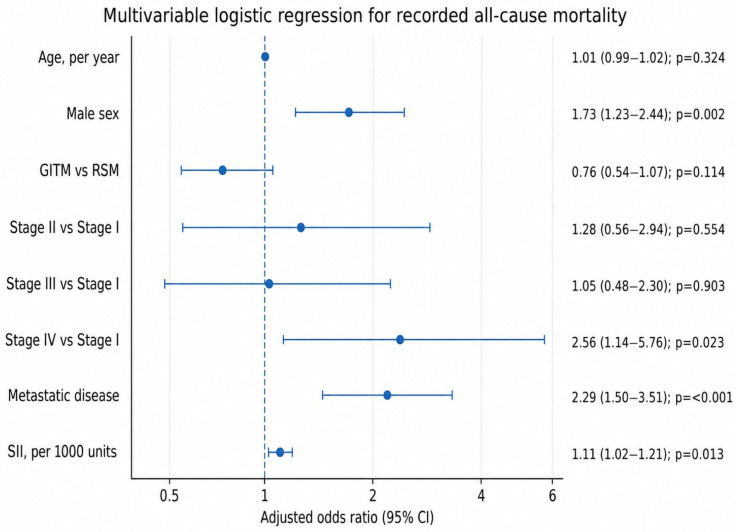
Forest plot of the multivariable logistic regression model for recorded all-cause mortality. Adjusted odds ratios and 95% confidence intervals are shown for age, sex, cancer type, cancer stage, metastatic disease, and SII. The model included age, sex, cancer type, stage, metastatic disease, and SII expressed per 1000-unit increase. The vertical dashed line indicates an odds ratio of 1.0.

After adding SII to the model (Model 2), male sex, stage IV disease, metastatic disease and SII remained associated with mortality. Each 1000-unit increase in SII was related to higher odds of death (OR 1.11, 95% CI 1.02–1.21; *p* = 0.013). The addition of SII slightly improved model discrimination, with AUC increasing from 0.719 in Model 1 to 0.729 in Model 2. The Hosmer–Lemeshow test did not indicate evidence of poor model fit for either model, with *p*-values of 0.840 for Model 1 and 0.162 for Model 2, respectively. The likelihood-ratio test indicated a statistically significant incremental contribution of SII (*p* = 0.009) (Figure 1).

We additionally evaluated the neutrophil-to-lymphocyte ratio (NLR) using the same multivariable model applied for SII. Higher NLR was independently associated with recorded all-cause mortality (OR 1.07 per one-unit increase, 95% CI 1.03–1.12; *p* < 0.001). The model including NLR had an AUC of 0.732, compared with 0.729 for the model including SII. The difference between the two models was small and not statistically significant (ΔAUC 0.0034, 95% CI −0.0023 to 0.0094; *p* = 0.250). Detailed results are presented in Supplementary Table S4.

### 3.3. Model Performance, Incremental Value, and Sensitivity Analyses

As a sensitivity analysis, SII was additionally evaluated according to tertiles. Mortality increased progressively across increasing SII tertiles, from 22.5% in the lowest tertile to 34.5% in the intermediate tertile and 47.4% in the highest tertile. After adjustment for age, sex, cancer type, stage, and metastatic disease, patients in the highest SII tertile had significantly higher odds of all-cause mortality compared with those in the lowest tertile (OR 1.93, 95% CI 1.30–2.86; *p* = 0.001). The intermediate tertile showed a non-significant trend toward higher mortality (OR 1.39, 95% CI 0.94–2.05; *p* = 0.099) (Supplementary Table S1).

**Figure 2:**
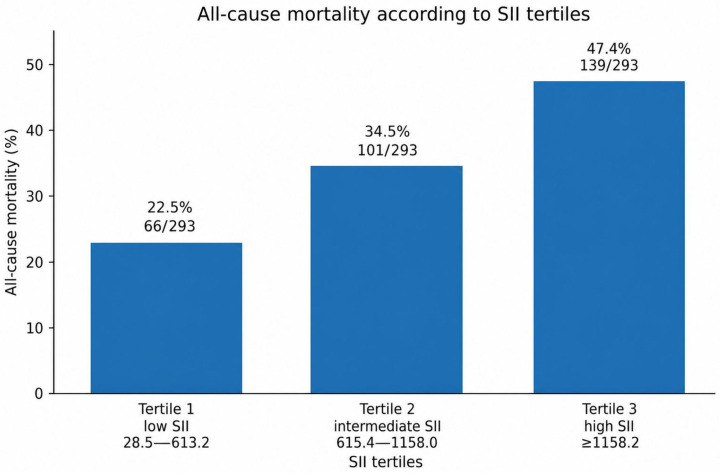
All-cause mortality according to SII tertiles in the overall cohort. Patients were ranked according to baseline SII values and divided into three equal-sized tertiles. Values above bars indicate mortality percentage and deaths/total patients. The displayed SII ranges represent the observed minimum and maximum values within each tertile.

Figure 2 illustrates the stepwise increase in all-cause mortality across SII tertiles. Mortality was lowest in patients with low SII and highest in those with high SII, increasing from 22.5% to 34.5% and 47.4%, respectively.

In an additional interaction analysis, no significant interaction was observed between SII and cancer type, suggesting that the association between SII and all-cause mortality did not statistically differ between RSM and GITM (Supplementary Table S2).

## 4. Discussion

In this retrospective cohort, patients with respiratory system malignancies had higher SII values than those with gastrointestinal tract malignancies. They also had a higher proportion of stage IV disease and higher recorded all-cause mortality. This pattern indicates that SII reflects the overall inflammatory and clinical burden of the patient, rather than a single isolated laboratory abnormality. This is in line with the concept that cancer-related inflammation depends on both tumor biology and the host response [11] and with the cardio-oncology perspective that cancer and cardiovascular disease share several risk factors and mechanisms, including smoking, obesity, diabetes, hypertension, dyslipidemia and chronic inflammation [12].

In our study, higher SII remained associated with recorded all-cause mortality after adjustment for age, sex, cancer type, tumor stage and metastatic disease. This is relevant because stage and metastasis are among the strongest determinants of outcome in patients with cancer. The persistence of the association after adjustment suggests that SII may capture part of the inflammatory and general clinical condition that is not fully described by stage and metastatic status alone. Similar findings have been reported in previous studies of solid tumors, although the strength of the association varies across tumor types and clinical settings [13].

The differences between RSM and GITM are also important. It should be noted that the RSM group was not restricted to lung cancer but also included malignancies of the larynx and bronchi. Likewise, the GITM group included biologically distinct tumors, including gastric, colorectal, hepatocellular, and pancreatic carcinomas. Patients with RSM were younger, more often male, more frequently had stage IV disease, and had higher mortality. They also had higher neutrophil and platelet counts, lower lymphocyte counts, and higher SII, indicating a more pronounced systemic inflammatory profile. Studies in lung cancer, including small-cell lung cancer and operable non-small-cell lung cancer, have also reported an association between SII and prognosis, and in some settings with disease stage [14,15,16]. In contrast, patients with GITM were older and had more cardiometabolic comorbidity, including diabetes, hypertension, and obesity. The apparently higher frequency of documented metastatic disease in the GITM group despite the higher proportion of stage IV disease among patients with RSM should be interpreted with caution, as metastatic disease was recorded only when explicitly documented in the medical records and may therefore reflect differences in retrospective documentation. Despite this, mortality was higher in the RSM group. In this cohort, tumor stage and systemic inflammation therefore appeared to carry more prognostic weight than cardiovascular comorbidity alone. Data from gastric and colorectal cancer also support a prognostic role of SII; however, the heterogeneity of the GITM group argues against assuming that SII has the same clinical meaning across all gastrointestinal malignancies [17,18,19,20].

The biological basis of these findings is plausible. SII combines neutrophils, lymphocytes, and platelets, all of which are involved in cancer-related inflammation. Neutrophilia may reflect systemic inflammation, tumor-related immune activation, infection, or stress response. Lymphopenia may indicate impaired immune reserve or poorer general condition. Platelets may contribute to inflammation, angiogenesis, metastatic spread, and the link between thrombosis, inflammation, and cancer [21]. A high SII may therefore be best understood as a combined signal of inflammation, immune imbalance, and tumor-related burden.

Although SII was associated with mortality, its incremental prognostic value was modest. The improvement in model discrimination after adding SII was small: the AUC increased from 0.719 to 0.729 and the likelihood-ratio test was statistically significant. This shows that SII adds information to the clinical model, but the practical magnitude of this improvement is limited. SII should therefore not be used as a standalone prognostic tool. It is more appropriate to regard it as an additional marker that supports clinical assessment together with stage, metastatic disease, age, sex and cancer type. This interpretation is consistent with meta-analyses showing prognostic relevance of SII in solid tumors without demonstrating that SII alone is sufficient for individual risk prediction [13].

From a clinical perspective, SII has practical advantages because it is inexpensive, easily calculated from routine blood counts, and widely available. Our findings suggest that SII may help identify patients with a higher systemic inflammatory burden and an increased risk of mortality. However, the modest improvement in model discrimination observed after adding SII indicates that its contribution to individual risk prediction is limited. In routine oncology practice, SII is therefore best viewed as a supportive marker that complements established prognostic factors, such as tumor stage, metastatic disease, and the patient’s overall clinical condition, rather than as a standalone tool for treatment decisions or risk stratification. Further prospective studies are needed to determine whether incorporating SII into routine clinical assessment improves patient management and outcomes.

The additional NLR analysis showed a similar pattern. NLR was independently associated with mortality and produced a slightly higher AUC than SII, but the difference in discrimination was small and not statistically significant. These findings suggest that NLR and SII provide broadly comparable prognostic information in this cohort. As the main objective of the study was to evaluate SII rather than to compare inflammatory biomarkers, SII remains the primary focus of the analysis.

The Hosmer–Lemeshow test did not indicate evidence of poor model fit for either the clinical model or the model including SII. However, this test alone does not provide a comprehensive assessment of calibration, and model performance in this retrospective cohort does not guarantee similar performance in other populations. External validation would therefore be required before either model could be considered for clinical prediction. The tertile analysis provided a clearer clinical picture of the association. Mortality increased stepwise from the lowest to the highest SII tertile, and patients in the highest tertile had significantly higher adjusted odds of death compared with those in the lowest tertile. The tertile-based results therefore confirmed the association observed in the continuous model and showed that patients with a greater systemic inflammatory burden had worse recorded outcomes.

The interaction analysis did not show a statistically significant interaction between SII and cancer type. Therefore, although SII values and mortality were higher in the RSM group, the study did not demonstrate that the association between SII and mortality differed significantly between RSM and GITM. SII should therefore be interpreted as a general mortality-associated inflammatory marker in this cohort, not as a marker specific to one tumor group.

In this cohort, SII was not clearly related to the combined cardiometabolic and cardiovascular burden. Although patients with a greater burden of cardiometabolic risk factors and cardiovascular disease had higher recorded all-cause mortality, SII values did not increase accordingly. This suggests that, in hospitalized oncology patients, SII primarily reflects systemic inflammation associated with the underlying malignancy and overall disease burden rather than the number of coexisting cardiometabolic and cardiovascular conditions. Because our outcome was recorded all-cause mortality in a cancer population, the underlying malignancy, tumor stage, and metastatic disease were likely the major determinants of overall mortality. This should be taken into account when comparing our findings with studies from non-oncological populations, where SII has mainly been evaluated in relation to cardiovascular outcomes [22,23].

This study has several limitations. It was a retrospective single-center study, which limits causal interpretation and generalizability. Another important limitation is the broad grouping of malignancies into respiratory and gastrointestinal categories. Both groups included tumors with different anatomical origins, histological characteristics, etiological backgrounds, and clinical behavior. The study was not designed to provide separate adjusted analyses for individual tumor entities or histological subtypes. Dividing the cohort into multiple smaller subgroups, including gastric, colorectal, hepatocellular, pancreatic, and respiratory tumor subtypes, would have reduced the number of patients and events available for each multivariable model and could have produced unstable estimates. The findings should therefore be interpreted at the level of the two broad organ-system groups and should not be directly extrapolated to individual malignancies. Mortality was analyzed as a recorded binary outcome because complete time-to-event data were not available for all patients, so the results should be interpreted as associations with recorded all-cause mortality rather than survival estimates. SII was calculated from a single baseline blood sample obtained during the first hospitalization before the initiation of treatment, and changes in inflammatory status during treatment or disease progression could not be assessed. Performance status, active infection, corticosteroid exposure, anticoagulation, and detailed information on subsequent systemic anticancer therapy were not systematically available. Although baseline laboratory parameters were obtained before the initiation of systemic anticancer therapy, differences in subsequent treatment may have influenced all-cause mortality and could not be accounted for in the present analysis. Although the models were adjusted for the main demographic and cancer-related factors available in our dataset, we cannot exclude the influence of other factors that were not measured or were incompletely recorded. The findings should therefore be interpreted as associations rather than evidence of a causal relationship.

Information regarding recent surgical interventions and the exact interval between surgery and baseline blood sampling was not systematically available. Although, according to routine institutional practice, patients are generally referred for systemic oncological treatment approximately one month after surgery and following adequate postoperative recovery, a residual influence of recent surgery on inflammatory parameters, including SII, cannot be completely excluded.

The study also has strengths. It includes a real-world cohort of hospitalized cancer patients, compares two clinically relevant tumor groups, and evaluates SII together with major oncological determinants of outcome. The analysis includes multivariable adjustment, model discrimination and calibration, nested likelihood-ratio testing, tertile-based sensitivity analysis, and interaction testing. Overall, these elements support a cautious interpretation of SII as a simple inflammatory marker associated with mortality, without overstating its role as an independent predictive tool.

## 5. Conclusions

In conclusion, at the level of the broad organ-system groups analyzed, SII was higher in patients with respiratory system malignancies than in those with gastrointestinal tract malignancies and was associated with recorded all-cause mortality in the overall cohort. The association was consistent in sensitivity analysis, while the addition of SII produced only a modest improvement in model performance. These findings support the use of SII as an accessible marker of systemic inflammatory burden, but its interpretation should remain tied to tumor stage, metastatic disease, and the broader clinical context.

## Figures and Tables

**Table 1:** Baseline characteristics according to cancer type.

Characteristic	RSM (n = 350)	GITM (n = 529)	*p*-Value
Age, years	62.5 [56.0–69.0]	68.0 [61.0–74.0]	<0.001
Male sex	282/350 (80.6%)	303/529 (57.3%)	<0.001
Metastatic disease	74/350 (21.1%)	143/529 (27.0%)	0.057
Stage, overall distribution			<0.001
Stage I	11/350 (3.1%)	37/529 (7.0%)	
Stage II	33/350 (9.4%)	112/529 (21.2%)	
Stage III	96/350 (27.4%)	209/529 (39.5%)	
Stage IV	210/350 (60.0%)	171/529 (32.3%)	
All-Cause Mortality	148/350 (42.3%)	158/529 (29.9%)	<0.001

Continuous variables are presented as median [interquartile range], and categorical variables as n/N (%). *p*-values were calculated using the Mann–Whitney U test for continuous variables and chi-square test for categorical variables.

**Table 2:** Clinical characteristics according to cancer type.

Clinical Characteristic	RSM (n = 350)	GITM (n = 529)	*p*-Value
Coronary artery disease	23/350 (6.6%)	26/528 (4.9%)	0.373
Myocardial infarction	15/350 (4.3%)	19/529 (3.6%)	0.731
Stroke	26/350 (7.4%)	15/529 (2.8%)	0.003
Diabetes mellitus	33/350 (9.4%)	103/529 (19.5%)	<0.001
Hypertension	172/350 (49.1%)	320/529 (60.5%)	0.001
Obesity	50/350 (14.3%)	115/529 (21.7%)	0.007
Atrial fibrillation	27/350 (7.7%)	61/529 (11.5%)	0.083
Dyslipidemia	21/350 (6.0%)	30/529 (5.7%)	0.955
Vascular disease	12/350 (3.4%)	15/529 (2.8%)	0.765
Valvular disease	7/350 (2.0%)	20/529 (3.8%)	0.194
Heart failure	26/350 (7.4%)	40/529 (7.6%)	1.000
Smoking	173/350 (49.4%)	128/529 (24.2%)	<0.001
Alcohol use	55/350 (15.7%)	77/529 (14.6%)	0.708
Pulmonary embolism	7/350 (2.0%)	13/529 (2.5%)	0.830
Asymptomatic pulmonary embolism	0/350 (0.0%)	5/529 (0.9%)	0.163
Deep vein thrombosis	10/350 (2.9%)	32/529 (6.0%)	0.044

Categorical variables are presented as n/N (%). After cleaning, one GITM value for Coronary artery disease was missing; percentages are based on available data. *p*-values were calculated using the chi-square test or Fisher exact test where appropriate.

**Table 3:** Laboratory parameters and inflammatory indices according to cancer type.

Parameter	RSM (n = 350)	GITM (n = 529)	*p*-Value
Neutrophils	5.9 [4.4–7.8]	4.6 [3.5–6.1]	<0.001
Lymphocytes	1.5 [1.0–2.0]	1.7 [1.2–2.1]	<0.001
Platelets	278.5 [211.0–367.0]	256.0 [203.0–338.0]	0.005
Systemic immune-inflammation index (SII)	1117.8 [661.1–2051.2]	716.8 [456.9–1146.8]	<0.001

Continuous variables are presented as median [interquartile range]. *p*-values were calculated using the Mann–Whitney U test.

**Table 4:** Multivariable logistic regression models for all-cause mortality.

Variable	Model 1 OR (95% CI)	*p*-Value	Model 2 + SII OR (95% CI)	*p*-Value
Age, per year	1.01 (0.99–1.02)	0.316	1.01 (0.99–1.02)	0.324
Male sex	1.68 (1.20–2.36)	0.003	1.73 (1.23–2.44)	0.002
GITM vs. RSM	0.71 (0.50–1.00)	0.047	0.76 (0.54–1.07)	0.114
Stage II vs. Stage I	1.34 (0.59–3.07)	0.486	1.28 (0.56–2.94)	0.554
Stage III vs. Stage I	1.08 (0.49–2.36)	0.849	1.05 (0.48–2.30)	0.903
Stage IV vs. Stage I	2.77 (1.24–6.21)	0.013	2.56 (1.14–5.76)	0.023
Metastatic disease	2.36 (1.54–3.59)	<0.001	2.29 (1.50–3.51)	<0.001
SII, per 1000 units	-	-	1.11 (1.02–1.21)	0.013
**Model performance, calibration, and incremental value**				
N/events	879/306		879/306	
AUC	0.719		0.729	
Hosmer–Lemeshow *p*-value	0.840		0.162	
Likelihood-ratio test for adding SII	Reference		*p* = 0.009	

Footnote: Model 1 included age, sex, cancer type, stage, and metastatic disease. Model 2 included the same covariates plus SII expressed per 1000 units. OR—odds ratio; CI—confidence interval; SII—systemic immune-inflammation index; AUC—area under the receiver operating characteristic curve. Model calibration was assessed using the Hosmer–Lemeshow goodness-of-fit test. The incremental value of SII was assessed by nested likelihood-ratio testing comparing Model 2 against Model 1.

## Data Availability

The datasets used and analyzed during the current study are available from the corresponding author on reasonable request.

## Supplementary Materials

The following supporting information can be downloaded at https://www.mdpi.com/article/10.3390/jcm15145752/s1. Table S1: Sensitivity analysis using SII tertiles; Table S2: Interaction analysis between SII and cancer type for all-cause mortality; Table S3: Association of combined cardiometabolic and cardiovascular burden with SII and mortality; Table S4: Comparison of the prognostic performance of SII and NLR for recorded all-cause mortality.

## Abbreviations

The following abbreviations are used in this manuscript:

AF	atrial fibrillation
AUC	area under the receiver operating characteristic curve
CAD	coronary artery disease
CI	confidence interval
IQR	interquartile range
DVT	deep vein thrombosis
GITM	gastrointestinal tract malignancies
HF	heart failure
MI	myocardial infarction
NHIF	national health insurance fund
NLR	neutrophil-to-lymphocyte ratio
OR	odds ratio
PE	pulmonary embolism
ROC	receiver operating characteristic
RSM	respiratory system malignancies
SII	systemic immune-inflammation index
