# Supplementary material for: Respiratory Versus Gastrointestinal Malignancies: Systemic Inflammation, Cardiovascular Burden, and All-Cause Mortality
Source: J Clin Med. 2026 Jul 22;15(14):5752. doi: 10.3390/jcm15145752 (PMC13413368; doi:10.3390/jcm15145752)
Supplement: Supplementary file 1 [file jcm-15-05752-s001.zip › jcm-4433694-supplementary.pdf]

Supplementary

Supplementary Table S1. Sensitivity analysis using SII tertiles

SII category	SII, median [IQR]	n	Deaths	Mortality	Adjusted OR (95% CI)	p-value
Tertile 1 — low SII	411.8 [302.8–525.8]	293	66	22.5%	Reference	—
Tertile 2 — intermediate SII	871.5 [718.6–998.9]	293	101	34.5%	1.39 (0.94–2.05)	0.099
Tertile 3 — high SII	2060.0 [1476.6–3028.7]	293	139	47.4%	1.93 (1.30–2.86)	0.001
Ordinal trend per tertile increase	—	879	306	34.8%	1.39 (1.14–1.69)	0.001

Abbreviations: SII, systemic immune-inflammation index; IQR, interquartile range; OR, odds ratio; CI, confidence interval. Adjusted ORs were derived from multivariable logistic regression models adjusted for age, sex, cancer type, stage, and metastatic disease. The lowest SII tertile was used as the reference category.

Interpretation: Mortality increased progressively across SII tertiles. Patients in the highest SII tertile had significantly higher adjusted odds of all-cause mortality compared with those in the lowest tertile, supporting the robustness of the continuous SII analysis.

Supplementary Table S2. Interaction analysis between SII and cancer type for all-cause mortality

Variable / comparison	Adjusted OR (95% CI)	p-value
SII, per 1000 units in RSM	1.11 (0.99-1.24)	0.066
SII x GITM interaction	1.01 (0.85-1.20)	0.916
Estimated SII effect in GITM	1.12 (0.98-1.28)	0.093
Likelihood-ratio test for interaction	-	0.916

Abbreviations: SII, systemic immune-inflammation index; OR, odds ratio; CI, confidence interval. The model included age, sex, cancer type, stage, metastatic disease, SII per 1000 units, and the SII x cancer type interaction term. RSM was used as the reference cancer type.

Note: The p-values for the cancer-type-specific SII estimates are derived from the interaction model and reflect conditional estimates within RSM or GITM, whereas the p-value in the main model refers to the overall SII association without an interaction term.

Interpretation: The interaction term was not statistically significant, indicating no evidence that the association between SII and all-cause mortality differed significantly between RSM and GITM groups.

Supplementary Table S3. Association of combined cardiometabolic and cardiovascular burden with SII and mortality

Combined burden category	n	Deaths	Mortality	SII, median [IQR]
0–1 recorded conditions	413	118	28.6%	827.6 [515.8–1416.0]
2–3 recorded conditions	340	134	39.4%	890.9 [539.1–1538.7]
≥4 recorded conditions	126	54	42.9%	905.1 [527.2–1636.2]
p-value			0.001	0.597

Abbreviations: SII, systemic immune-inflammation index; IQR, interquartile range; The combined burden was calculated as the total number of recorded cardiometabolic risk factors, cardiovascular diseases, and related clinical conditions documented in the medical records. Mortality across burden categories was compared using the chi-square test. SII values across categories were compared using the Kruskal–Wallis test. The Spearman correlation between SII and the combined burden count was weak and non-significant (rho = 0.037; p = 0.276).

Supplementary Table S4. Comparison of the prognostic performance of SII and NLR for recorded all-cause mortality.

Variable	Model with SII OR (95% CI)	p-value	Model with NLR OR (95% CI)	p-value
Age, per year	1.01 (0.99–1.02)	0.324	1.01 (0.99–1.02)	0.321
Male sex	1.73 (1.23–2.44)	0.002	1.71 (1.22–2.41)	0.002
GITM vs. RSM	0.76 (0.54–1.07)	0.114	0.75 (0.53–1.06)	0.106
Stage II vs. Stage I	1.28 (0.56–2.94)	0.554	1.29 (0.56–2.96)	0.547
Stage III vs. Stage I	1.05 (0.48–2.30)	0.903	1.06 (0.48–2.31)	0.895
Stage IV vs. Stage I	2.56 (1.14–5.76)	0.023	2.54 (1.13–5.71)	0.024
Metastatic disease	2.29 (1.50–3.51)	<0.001	2.30 (1.50–3.52)	<0.001
Inflammatory marker	SII: 1.11 (1.02–1.21), per 1000 units	0.013	NLR: 1.07 (1.03–1.12), per 1-unit increase	<0.001
AUC	0.729	—	0.732	—
Likelihood-ratio test vs. clinical model	6.91	0.009	13.48	<0.001

Abbreviations: SII, systemic immune-inflammation index; NLR, neutrophil-to-lymphocyte ratio; OR, odds ratio; CI, confidence interval; AUC, area under the receiver operating characteristic curve. Both models were adjusted for age, sex, cancer type, stage, and metastatic disease. The clinical model without an inflammatory marker had an AUC of 0.719. The likelihood-ratio tests compare each inflammatory-marker model with the clinical model. Bootstrap comparison of model discrimination showed ΔAUC (NLR − SII) = 0.0034 (95% CI −0.0023 to 0.0094; p=0.250).

Interpretation: Both SII and NLR improved the clinical model. Although the model including NLR had a marginally higher AUC, the bootstrap comparison showed no statistically significant difference in discrimination between the two models.
